# Surgery

**Published:** 1894-09

**Authors:** W. H. Harsant


### SURGERY.

The question of the best mode of operating for hemorrhoids has long exercised the minds of surgeons, and it is curious to note the fluctuation of opinion which has taken place at different periods on this subject. Hippocrates strongly recommended the ligature, and this plan was followed by Paulus Ægineta. Dupuytren was one of the first to recommend excision, and he was in the habit of practising this method in all his cases, but as he frequently met with troublesome hemorrhage he recommended that a cautery iron should be applied to the raw surface in all cases where bleeding took place.[1] Sir Astley Cooper was at first an advocate for excision; but having the misfortune to lose several patients from hemorrhage, he altered his views and recommended ligature.[2] Henry Smith,[3] by the introduction of his clamp, did much to popularise the use of the cautery, and the discovery of the handy nature of Paquelin's benzoline cautery served still further to render this method of treatment the one universally employed. As in so many other departments of surgery, however, the pendulum has now swung back again, and at the present time excision is probably the most popular method in use, and for this we are indebted in great measure to Whitehead of Manchester, who has done much to improve the operation, especially by pointing out how to avoid hemorrhage, and to secure primary union.[4] His description of the operation is as follows:—(1) The patient, previously prepared for the operation and under the complete influence of an anæsthetic, is placed on a high narrow table in the lithotomy position, and maintained in this position either by a couple of assistants or by Clover's crutch. (2) The sphincters are thoroughly paralysed by digital stretching, so that they have no "grip," and permit the hemorrhoids and any prolapse there may be, to descend without the slighest impediment. (3) By the use of scissors and dissecting forceps the mucous membrane is divided at its junction with the skin round the entire circumference of the bowel, every irregularity of the skin being carefully followed. (4) The external and the commencement of the internal sphincters are then exposed by a rapid dissection, and the mucous membrane and attached hemorrhoids, thus separated from the submucous bed on which they

[1] *Lesions of the Vascular System*, Syd. Soc., 1854, p. 126.

[2] *Principles and Practice of Surgery*, 1837, vol. i., p. 432.

[3] *Lancet*, 1878, vol. i., p. 561. [4] *Brit. M. J.*, 1887, vol. i., p. 449.

rested, are pulled bodily down, any undivided points of resistance being snipped across, and the hemorrhoids brought below the margin of the skin. (5) The mucous membrane above the hemorrhoids is now divided transversely in successive stages, and the free margin of the severed membrane above is attached, as soon as divided, to the free margin of the skin below by a suitable number of sutures. The complete ring of pile-bearing mucous membrane is thus removed.

Three hundred operations are reported by Whitehead without a death or a single instance of secondary hemorrhage.

Dr. Arpad G. Gerster records his experience at the Mount Sinai Hospital,[1] and mentions 266 operations for hemorrhoids, of which 156 were treated by the clamp and cautery, 63 by ligature, and 47 by Whitehead's operation. He decidedly prefers the clamp and cautery for the majority of cases requiring operation, but approves of Whitehead's method for severe cases.

At the New York Post-graduate Hospital[2] the following advantages are claimed for clamp and cautery over all other methods for radical cure of hemorrhoids :—(1) The least pain. (2) The quickest recovery. (3) The least vesical and general disturbance.

Dr. Kelsey has used this method exclusively for the last ten years without ever having a serious accident or complication of any sort. "This statement is made simply to offset the statement made by Allingham years ago, that the clamp and cautery is at least six times as fatal as the ligature—a statement frequently repeated, as often challenged, and for which neither of the Allinghams seem ready to furnish any proof, in spite of the fact that when last repeated (by which time the figure had grown to eight instead of six) three public requests for proof appeared almost simultaneously in British Journals."

Dr. Kelsey has operated upon 150 cases without a single case of hemorrhage or failure to cure, and with only one case in which the subsequent use of a bougie for contraction was necessary. The patients were generally allowed up on the third day, and many of them left hospital on the fourth or fifth day. He sums up his personal experience as follows :—

"If you wish to radically cure your patients with the least possible pain, loss of time, confinement to the house, and risk of accidents—use the clamp and cautery.

"If you wish to accomplish the same result with no more risk, but with more pain, more local and general disturbance, and longer confinement—use the ligature.

"If you wish to take an hour to do what can as well be done in a minute, and gain nothing by it in results—use Whitehead's operation."

There are many cases of carcinoma of the rectum which are too high for removal from the perineum, and too low for removal

[1] *Tr. Am. Surg. Ass.*, vol. xi., 1893, p. 130. [2] *N. York M. J.*, vol. lix., 1894, p. 750.

by cœliotomy. Dr. Kraske,[1] of Freiburg, communicated to the German Surgical Congress a method which he had first worked out on the cadaver, and then tried on the living subject. An incision is first made in the soft parts in the middle line from the second sacral vertebra to the anus, dividing the muscular attachments to the sacrum as far as the ends of the bone on the left side; the coccyx is then excised and a portion of the lower end of the sacrum cut away with bone forceps; the attachments of the two sacro-sciatic ligaments are next divided from the sacrum, and room thus made to reach the rectum above the levator ani muscle. Or the sacrum may be cut through transversely at the level of the third sacral foramen, and the sacro-sciatic ligaments cut through on both sides, when room will be found to introduce the whole hand into the pelvis.

This operation, now widely known as Kraske's operation, has opened up a large field of operative measures for cancer of the rectum, and is now being adopted very freely by German and American surgeons. It has not yet been practised extensively in this country, but the accounts given of it by those who have tried it are for the most part favourable.

Five cases of extirpation of rectum are recorded by Gerster,[2] four being for carcinoma, and one for stricture caused by ulcerative proctitis. Of these five patients, two died in consequence of the operation, both of acute anæmia; one suffering from carcinoma, the other from ulcerative proctitis. In both Kraske's operation was done. Two other cases were successful, and also one case of removal of the rectum by the old-fashioned perineal method. He advises a preliminary colotomy in all cases where much fæcal distress exists, and also insists upon the advantage of the prone position of the patient, with the breech well elevated by hard cushions.

Pilcher[3] mentions six operations with two deaths. Ball[4] gives four cases, all successful.

A remarkably successful case of excision of the rectum for cancer is reported by Dr. H. O. Marcy.[5] The growth was situated two inches above the anus and extended as far as the index finger could reach. Iuguinal colotomy was first performed, and then a month later the diseased gut was excised by a modification of Kraske's operation. An incision was made posteriorly, commencing about an inch from the anal outlet, and carried upwards on the median line upon the sacrum. The coccyx and two-fifths of the sacrum were removed, allowing room to dissect the rectum from its attachment, dividing posteriorly the meso-rectum and entering at once into the peritoneal cavity. The rectum was now divided two inches above the anus, and the constricting diseased portion split open upon its posterior border for the double purpose of ascertaining

[1] *Ann. Surg.*, vol. ii., 1885, p. 415. [2] *Tr. Am. Surg. Ass.*, vol. xi., 1893, p. 143.
[3] *Ibid*, p. 150. [4] *The Rectum and Anus*, 2nd edit., 1894, p. 375.
[5] *Mathews' M. Quart.*, vol. i., 1894, p. 80.

the limitation of the disease and to aid thereby the careful separation of the bowel from its anterior attachments, which to the base of the bladder were everywhere pronounced. The rectum was then divided above the growth, the disease having invaded about four inches of the bowel.

Dr. Marcy took advantage of a suggestion by Dr. H. O. Walker, of Detroit, and made use of a large-sized Murphy button which he had had specially made to hold together the divided ends of the intestine. The use of this button was most satisfactory. Complete union of the gut eventually took place, although a sinus remained for a short time. The button was removed on the twelfth day with a little difficulty, not having freed itself quite entirely from its attachment; but its removal was hastened because pressure of the button upon the neck of the bladder caused discomfort and some difficulty in urination. The patient was discharged from the hospital on the twentieth day, and when seen three months afterwards remained free from disease and in improved health. The inner portion of the bowel was marked by an elastic constricting ring, readily admitting the finger.

W. H. Harsant.

